# Intubation of the Larynx in Diphtheria

**Published:** 1907-07-20

**Authors:** 


### POINTS IN TREATMENT

### INTUBATION OF THE LARYNX IN DIPHTHERIA.

THE question often arises whether to intubate the larynx in cases of diphtheria in which there is considerable obstruction to the airway, or to perform tracheotomy either at once or presently if it should be necessary. No arbitrary answer can be given to the question; each practitioner forms his own judgment for himself.

The arguments in favour of intubation are mainly four:—

1. Intubation is not a "cutting operation," and therefore will be allowed by many parents who would object to their child being "cut"; besides which, if it enables tracheotomy to be avoided, it leaves no wound which runs the risk of going septic, which takes time to heal, and which leaves a disfiguring scar—a point of considerable importance in the case of a girl who is likely to appear much in society in future days.

2. Intubation can be performed at an earlier stage of the dyspnœa than that at which tracheotomy is definitely indicated as the only alternative.

3. It is generally stated that the after-histories of persons who recover from diphtheria for which tracheotomy has been performed are bad; that these patients seem particularly liable to subsequent lung trouble—bronchopneumonia in the near future, phthisis later. The reason appears to be that inhalation of unwarmed and unmoistened air into the lungs through a tracheotomy wound irritates and damages the pulmonary tissues, which are accustomed to receive the air both warmed and moistened in its transit through the upper air passages. It is argued that, with intubation, the air inhaled is natural in these respects, and that therefore the after-effects as regards the lungs are better.

4. It sometimes happens that, after tracheotomy, there is a great deal of difficulty in leaving out the tube; some of the factors concerned in this have been discussed in a former issue (June 15, p. 282), and we gave the opinion that in many instances the trouble was due to injury to, or division of, the cricoid cartilage at the time the tracheotomy was performed; if this be so it is an avoidable difficulty, and when the cricoid cartilage is uninjured and the attempt to leave the tube out is not postponed beyond forty-eight hours after the operation, the proportion of cases in which the tube cannot soon be dispensed with is very small.

On the other hand:—

1. Intubation is not by any means easy of performance without a good deal of practice; it is easy enough to do upon the cadaver, but quite otherwise in the living dyspnœic child. The struggles of the latter aggravate the dyspnœa unless the intubation is quickly accomplished; to those who are not already accustomed to doing either, tracheotomy must necessarily be the procedure of election.

2. There is a certain amount of risk that the delicate structures of the glottis may be considerably injured in the attempts to intubate; even if the actual manipulations do not damage the parts, the presence of the metal tube in contact with the inflamed soft tissues is liable to lead to further inflammation, or even to ulceration, perichondritis, and similar local troubles.

3. It often happens that the membranous exudate and surrounding œdema extend lower down than the intubation tube can reach; the result is that in a great many patients in whom intubation is first tried tracheotomy becomes inevitable a few hours later, and the prognosis is made considerably worse by the necessity for the double operation.

4. There is a great risk that the child may cough out the intralaryngeal tube. This is probably the most important objection of all; for whereas the nurse in attendance can fairly easily replace a tracheotomy tube, or can at least keep the orifice of the wound dilated until such time as the doctor can be sent for, she is quite unable to use the intubation apparatus, and the child will very likely suffocate before the practitioner can arrive. Intubation may have sufficient advantages over tracheotomy to be preferable in hospitals where a medical officer is always on the spot ready to deal with the emergency, but in private practice the risks of accident to the tube are too great; the doctor has not time to stay with the patient hour after hour, and yet we think that it is scarcely justifiable to go away from a child who is in such a condition that suffocation would be the immediate result if the tube should be coughed out. The only condition in which there may be a strong indication for trying intubation seems to be when the dyspnœa, though considerable, is yet not sufficient to call for urgent tracheotomy; even then, however, it is essential that the medical man should remain near the patient the whole time until all danger of suffocation is past, and he can remain with the patient just as well without performing intubation as he can after doing it. In private practice, where the patient must be left because of the necessities of other patients whom the doctor is bound to visit, tracheotomy is a very great deal safer than intubation. The worst statistics of the results of tracheotomy are those of cases where the operation has been postponed until the very latest possible moment; when tracheotomy is performed earlier the prognosis is by no means so bad. It is true that, in making it a general rule to perform the operation when laryngeal obstruction is only severe and not practically complete, a certain number of children will be operated upon who might have survived without it if they could have been continuously watched with all the instruments ready for the operation at a moment's notice; but, on the other hand, a great many lives are lost by trying to postpone tracheotomy. In the best interests of the patients, to whom it is a question of life or death, it is a great deal better to perform tracheotomy too early than too late; and we feel sure that, unless the doctor is able to remain at the bedside for many hours at a stretch, tracheotomy is greatly preferable to intubation in private practice.

